# Patients´ experiences of TENS as a postoperative pain relief method in the post-anesthesia care unit after laparoscopic cholecystectomy: a qualitative study

**DOI:** 10.1186/s12871-024-02872-4

**Published:** 2025-01-09

**Authors:** Eva Angelini, Charlotta Josefsson, Cecilia Ögren, Paulin Andréll, Axel Wolf, Mona Ringdal

**Affiliations:** 1 Institute of Health and Care Sciences, Sahlgrenska Academy, University of Gothenburg, Gothenburg, Sweden; 2 Department of Anesthesiology and Intensive Care Medicine, The West Hospitals, Region Västra Götaland, Sweden; 3 Department of Anaesthesiology and Intensive Care Medicine/Paincenter, Sahlgrenska University Hospital Östra, Region Västra Götaland, Gothenburg, Sweden; 4 Department of Anesthesiology and Intensive Care Medicine, Institute of Clinical Sciences, Sahlgrenska Academy, University of Gothenburg, Gothenburg, Sweden; 5 Institute of Nursing and Health Promotion, Oslo Metropolitan University, Oslo, Norway

**Keywords:** Laparoscopic cholecystectomy, Postoperative pain, PACU, TENS

## Abstract

**Background:**

High-frequency, high-intensity transcutaneous electrical nerve stimulation (HFHI TENS, i.e. 80 Hz and 40–60 mA) is an effective, fast-acting pain relief modality after elective surgery, offering pain relief within 5 min. Few studies have explored patients’ perspectives on using TENS in the post-anesthesia care unit. This study investigates patients’ experiences and perceptions of TENS as a complementary approach to traditional pharmacological pain management in postoperative care.

**Method:**

Patients undergoing elective laparoscopic cholecystectomy were offered TENS as an alternative to conventional pain treatment with IV opioids. Twenty participants attended telephone semi-structured telephone interviews a median of 12 days after surgery. Data were analysed using a thematic analysis according to Braun and Clark.

**Results:**

Participants expressed that TENS provided reassurance and relaxation, calmed them, and gave them a sense of control over their pain. Participants perceived a greater degree of autonomy as TENS could be administered independently. They conveyed a preference for TENS, which they experienced as a safe and fast-acting alternative to opioids, despite its limitations in managing severe pain and rapid offset upon discontinuation.

**Conclusion:**

To our knowledge, this is the first study that describes patients’ views on managing postoperative pain using TENS in the post-anesthesia care unit. This study indicates that patients desire alternatives to drugs for pain control in the postoperative setting. TENS has advantages, such as a rapid onset and offset and supporting patient autonomy, as well as drawbacks, such as being ineffective when pain is too severe. TENS could be included within the routine multimodal analgesia framework for person-centred postoperative pain management.

**Trial registration:**

The participants in the current study were retrospectively registered and recruited from a randomized controlled trial (RCT; registered at ClinicalTrials.gov: NCT04114149).

**Supplementary Information:**

The online version contains supplementary material available at 10.1186/s12871-024-02872-4.

## Introduction

In Western countries, gallstone disease frequently necessitates laparoscopic gallbladder removal that is typically performed in day surgery. A significant number of patients experience postoperative pain, often of severe intensity, which can delay discharge from the hospital and recovery, increase the risk of complications, and potentially evolve into chronic pain [1–4]. The conventional management of postoperative pain typically includes a range of analgesics, such as opioids, within a multimodal analgesia framework [5, 6]. Whilst effective, opioids are associated with adverse effects like nausea and sedation, and there is a concern over the risk of prolonged post-surgery opioid usage [7–10]. Since the majority of laparoscopic cholecystectomies are performed in outpatient settings, postoperative complications like pain and nausea are more debilitating than in a traditional hospital setting. Pain and nausea delay rehabilitation and discharge and can create situations where admission to in-patient care is the only solution, which is inconvenient for the patient and adds costs for the healthcare [11].

Transcutaneous electrical nerve stimulation (TENS) is a safe, effective modality for treating acute and chronic pain [12, 13]. High-frequency, high-intensity TENS (HFHI TENS, i.e. 80 Hz and 40–60 mA) is effective for postoperative pain relief after elective surgery in the post-anesthesia care unit (PACU) and offers fast pain relief within 5 min in treatment responders [14–17]. If a patient does not respond to TENS, conventional pharmacological treatment can be offered without delaying pain relief. TENS has few contraindications and no known serious adverse events [18]. After a brief instruction session, patients can manage the device themselves in the postoperative setting, thereby supporting patient autonomy and promoting person-centred care (PCC). The Gothenburg Centre for Person-Centred Care has developed an evidence-based framework for PCC, which emphasizes each patient’s personal narrative as the foundation for a collaborative partnership with healthcare providers. The primary aim of PCC is to engage and empower patients through shared decision-making principles [19, 20].

Despite the potential of HFHI TENS as a postoperative pain relieving modality that aligns with the principles of PCC, research into patients’ experiences of this stimulation regime remains limited. This study aims to investigate patients’ experiences and perceptions of HFHI TENS for postoperative pain management after elective laparoscopic cholecystectomy, focusing on its role as a complementary approach to traditional pharmacological treatment in postoperative care.

## Materials and methods

### Study design, setting and participants

This descriptive, qualitative study involved patient interviews and was undertaken at two county hospitals in Southwest Sweden. The hospitals perform general surgery in Sweden’s second-largest healthcare region (Västra Götaland). The study adheres to the Consolidated Criteria for Reporting Qualitative Research (COREQ) guidelines [21] (Supplementary Material Supplement 1).

The participants in the current study were recruited using convenience sampling from a randomized controlled trial (RCT; registered at ClinicalTrials.gov: NCT04114149). Patients who had undergone elective laparoscopic cholecystectomy were randomized in the PACU to receive either standard treatment with intravenous (IV) opioids or HFHI TENS if they reported postoperative pain intensity rated ≥ 3 on the Numerical Rating Scale (NRS; range 0–10, where 0 equals no pain and 10 the worst pain imaginable). The exclusion criteria for the RCT were unwillingness to participate, age < 18 years, having an electronic implant, chronic pain or habitually using opioids, insufficient knowledge of the Swedish language, impaired sensibility over likely sites for electrode application, and known substance abuse. Patients were also excluded if surgery or anesthesia parameters deviated from the study protocol.

The standard treatment with IV opioids in the RCT was doses of 2–3 mg oxycodone IV, repeated if required. The mean opioid consumption was 11.0 morphine equivalents [22]. HFHI TENS was administered for 1 min at high frequency (80–120 Hz) and high intensity (40–60 mA) and repeated once if there was an unsatisfactory pain-relieving effect after the first stimulation. Patients randomized to HFHI TENS could switch to standard treatment with IV opioids if two 1-minute-long stimulations with HFHI TENS did not achieve adequate pain relief, defined as a pain intensity of < 3 on the NRS. This effect was evaluated approximately 5 min after the start of the stimulation regime. If TENS provided adequate pain relief, patients were invited to continue using TENS at a lower intensity of their choosing during their postoperative hospital stay and taught how to manage the device. The RCT compared time in the PACU, pain relief, consumption of opioids, and satisfaction with treatment between the two groups. The mean opioid consumption in the HFHI TENS group was 4.5 morphine equivalents compared with 11.0 in the opioid group (*p* < 0.001) [22].

RCT participants were preoperatively informed about the potential for post-RCT follow-up interviews regarding their experiences of TENS. The inclusion criteria for the current study were participation in the parent RCT, randomization to the HFHI TENS group in the RCT, and sufficient knowledge of the Swedish language.

### Data collection

The research group created a semi-structured interview guide through comprehensive discussions [23] with senior researchers (PA, AW, MR). Open-ended inquiries with the main question: How have you experienced the treatment with TENS, including follow-up questions, to elicit more detailed insights based on the provided answers (Supplementary Material Supplement 2). A pilot interview was conducted to test the interview guide. The researchers deemed that the interview guide was relevant to the study aim, and no further changes were made. The pilot interview was therefore included in the analysis.

Telephone interviews were conducted between March 2022 and February 2023 by the authors (EA and CJ) 2 weeks post-discharge (median 12 days, range 7–23). Data collection was continued until data saturation was achieved and no new information emerged.

The interviewers had no pre-existing relationships with the participants to avoid bias. All interviews were audio-recorded and transcribed verbatim.

### Data analysis

Data from interviews were analyzed using a thematic analysis according to Braun and Clark [24]. This is a qualitative analytical method for identifying and analyzing patterns (themes) within the data. To ensure trustworthiness and rigor, we adhered to the six phases of thematic analysis. First, the interview transcripts were read repeatedly with reflective notes made by the first and last authors (EA, MR) to identify possible patterns. Second, the data were coded into meaningful groups. Third, the codes were sorted into potential subthemes, analyzed, and combined into themes. The preliminary subthemes and themes were discussed critically by all authors. Discussions were conducted openly and occurred on multiple occasions until a consensus was achieved. Fourth, the subthemes and themes were reviewed and revised for internal homogeneity and external heterogeneity. Fifth, the subthemes and themes were refined and defined to identify their essence scope and content. Finally, the findings were summarized, and representative participant quotes were selected to provide evidence of each theme’s credibility. NVivo version 14 was used for data management.

The first and last authors are both experienced specialized nurses with extensive experience in pain management and had no prior association with the respondents. The research team comprised individuals from both clinical and scientific backgrounds, offering diverse perspectives during the data analysis stage and enhancing the credibility of the findings.

### Ethics

This study was conducted in accordance with the Declaration of Helsinki 1975, as revised in 2013 [25]. The Regional Ethics Review Board in Gothenburg and the Swedish Ethical Review Authority approved the study (registration number 954 − 18, supplementary applications registry numbers 2021–03306 and 2023-01979-02). All participants gave oral and written consent to participate after receiving oral and written information about the study.

## Results

Twenty-four participants were initially enrolled in the study. However, four participants were subsequently excluded due to various reasons, including staff non-compliance with the study protocol, reoperation on the first day after surgery, absence of informed consent, and language difficulties or impaired memory of their time in the PACU. Consequently, the final cohort consisted of 20 participants: 17 women and 3 men. The age range of participants was between 31 and 73 years, with a mean age of 48 years. Within this cohort, representation was balanced between the two hospital sites, with 9 participants from the first site and 11 from the second. Thirteen participants obtained adequate pain relief with TENS, while 7 participants required the additional administration of IV opioids during their PACU stay.

Interviews with the participants were conducted within a postoperative timeframe ranging from day 7 to 23, at an average of 13 days postoperatively. The duration of interviews varied from approximately 10 to 19 min, with an average duration of 15 min. Data analysis resulted in two main themes, each with two sub-themes, shown in Table 1. Representative participant quotes that illustrate each theme are shown in Table 2.

**Table 1:** Overview of themes and sub-themes

Theme I	Theme II
TENS offers control	Preference for TENS for pain relief
- TENS provides reassurance, induces relaxation, and calms - TENS increases patient autonomy	- TENS as a safe and rapid alternative to opioids - TENS has limitations in severe pain and when discontinued

### Theme I – TENS offers control

Within the realm of pain management, the concept of control emerged as a focal point among participants employing TENS for postoperative pain relief. Participants emphasized that exerting control over pain—rather than aiming for its total elimination—was of paramount importance.

#### TENS provides reassurance, induces relaxation, and calms

Participants expressed that having a sense of control made their pain more manageable and tolerable. This ability to manage pain underscored the importance of maintaining control of their situation. The rapid onset of the pain-relieving effect and the participants exerting control while using TENS contributed to a sense of being in charge (Quote 1, Table 2). While using TENS, participants reported feeling empowered, alert, and capable of mobility, including getting out of bed and attending to personal needs, such as using the restroom. This perception of control promoted a sense of safety and trust in TENS for pain management.

Another dimension of this theme was that pain management with TENS reduced participants’ fears and worries about experiencing pain. Managing pain through TENS provided reassurance, induced relaxation, and elicited a calming influence, thereby diverting attention from the sensation of pain.

The majority of participants felt they benefitted from TENS. They were satisfied with managing their postoperative pain using TENS and felt a sense of pride in managing their pain with TENS. On the other hand, it was disheartening when TENS did not provide effective pain relief, and a transition to conventional pharmaceutical treatment (opioids) was needed (Quote 2, Table 2). Nevertheless, knowing they had access to alternative pain treatments and the ability to interrupt TENS contributed further to the participants’ feelings of security.

#### TENS increases patient autonomy

The pivotal role of autonomy and control in enhancing pain management underscores the importance of safety and trust in TENS. The participants described the TENS device as user-friendly, noting that they could control and manage it independently, contributing to an increased feeling of autonomy and a sense of assured pain relief (Quote 3, Table 2). Initially when HFHI TENS was administered for 1 min (with one possible repetition) at high frequency (80–120 Hz) and high intensity (40–60 mA), the participants felt recognized and engaged in consultation and felt involved in the decision-making process. Thereafter, participants could operate TENS at a lower-intensity regime by themselves. Their dependence on healthcare personnel diminished since they were able to autonomously and proactively adjust the intensity of the stimulation to optimize pain relief.

### Theme II – preference for TENS for pain relief

Many participants had previous experiences of surgery and postoperative pain management using opioids that had not always been positive. The participants referred to previous pharmaceutical treatments using terms such as pills, strong painkillers, opioid-like painkillers, morphine, drugs, medication, OxyContin, and opioids, and two respondents referred to milder pain relievers, such as paracetamol. TENS was experienced as a safe and effective method for pain relief as no patients reported any adverse effects of the TENS intervention and it was seen as a viable alternative to these conventional pain management approaches.

#### TENS as a safe and rapid alternative to opioids

Participants highlighted numerous advantages of TENS, with the primary benefit being the avoidance of strong painkillers, particularly opioids. Opioids were disliked due to their potential for adverse effects and the risk of dependence (Quote 4, Table 2).

Whilst the sensation of TENS could be somewhat uncomfortable initially, reducing the stimulation intensity made the discomfort more manageable. When TENS was applied, it induced a “welcome pain” that alleviated postoperative pain without “chemical” side effects (Quote 5, Table 2).

TENS was perceived as being safer than opioids, feeling harmless, and allowing faster recovery compared to opioids. The benefits of remaining alert and avoiding drowsiness associated with opioids were also acknowledged. The majority of participants held a positive attitude towards TENS from the outset, even if it did not always result in pain relief (Quote 6, Table 2).

Thirteen participants achieved pain relief using TENS exclusively, while the remaining seven required supplementary IV opioids as TENS had no or little impact on their pain. Within this latter group, there was some disappointment voiced over the ineffectiveness of TENS (Quote 7, Table 2).

#### TENS has limitations in severe pain and when discontinued

The seven participants who had severe pain at the initiation of treatment described the efficacy of TENS as limited (Quote 8, Table 2). The prompt turn-off effect of TENS was highlighted both as a drawback and as a benefit. If TENS was terminated prematurely, there was a risk of breakthrough pain (Quote 9, Table 2). On the other hand, any discomfort ceased immediately upon disconnecting from the TENS device, presenting an advantage in contrast to oral medication, which requires waiting for the duration of effect to pass (Quote 10, Table 2).

Participants considered the usage duration of TENS too short, and many expressed a desire to take the TENS device home, which was not possible within the study’s framework (Quote 11, Table 2).

**Table 2:** Quotes

Theme	Subtheme	Quote number	Quote
I. TENS offers control	Provides reassurance, induces relaxation, and calms	1	“*And then I feel like I have more control. I don’t need to worry*,* and it’s probably a bit that it felt safe*,* and I didn’t have to worry about it hurting”*
		2	“*I was positively inclined to try it; I had already been asked before the operation and really wanted to give it a chance. I had also received information that I could get another type of pain relief that TENS didn’t provide… I would have two attempts… and therefore I had that as a backup in case it didn’t help completely.”*
	Increases patient autonomy	3	“*Yes*,* well you can ask*,* or you can adjust the intensity of it*,* so in that way*,* you can control a bit more than when you just get a pill*,* for example. I got that little (TENS)box so I could do it myself too”*
II.Preference for TENS for pain relief	TENS as a safe and rapid alternative to opioids	4	*“Avoiding opioids which greatly affect you when you take them*,* there’s a high risk of addiction since they are highly addictive*,* and there are side effects when taking such strong pain killers*,* and I think that’s a big advantage to avoid”*
		5	*“You don’t feel chemically affected even though you’ve had anesthesia and all that. It’s not the same feeling at all. I’ve had surgery once before*,* and this time it was so nice to just be able to get up and then quietly return to bed*,* lie down*,* and* switch on this”
		6	*“I would have preferred just having TENS because I also get very affected by the morphine*,* so I was hoping that I would be lucky enough to be randomized to TENS*,* which I did*,* but unfortunately*,* it didn’t have the desired effect that I needed at that moment”*
		7	*“I had a strong preference for TENS since morphine significantly affects me. I was hopeful about being assigned to the TENS group*,* which happened. Sadly*,* it didn’t provide the relief I was looking for at the time”*
	TENS has limitations in severe pain and when discontinued	8	“*There wasn’t really a choice because I had gotten really bad pain*,* and it (TENS) didn’t work”*
		9	“*The downside was when it was turned off afterwards*,* then it hurt terribly. I had so much pain that it made me throw up”*
		10	“*The advantage is that you can control it a bit more than if you just take some other medication that you just swallow. Because if you get any other side effects from medication*,* you have to wait until they wear off*”
		11	“*I would have preferred that option (TENS) over taking opioids. Now I was given opioids to take home*,* but I didn’t need to take them. I managed without and just took paracetamol* ”

## Discussion

This interview study elucidated several aspects of patient experiences of HFHI TENS as a safe intervention to treat postoperative pain after laparoscopic cholecystectomy. The results highlight the importance of patients remaining in control in a vulnerable situation and underscore their reluctance to use pharmacological treatments for pain relief in the PACU setting.

Postoperative pain is a distressing concern for patients. Many patients feel a measure of anxiety in handing over control of pain management to healthcare providers (2). The first theme identified in our study was the importance of being allowed to make an active decision regarding how one’s pain relief is managed. TENS offered the possibility to exert control over one’s pain, and this aspect of treatment was regarded with nearly equal importance to the actual pain relief itself. For the participants, having a sense of control made their pain more manageable. Moreover, knowing that if TENS did not alleviate pain, one could switch over to standard treatment with opioids contributed to a sense of safety and promoted trust in healthcare providers.

Patients were taught to manage the TENS unit by health care providers in the PACU, to adjust the intensity (i.e. lower the mA used) and the electrode placement as needed. The lower-intensity TENS regime that patients could administer themselves when needed after a successful high-intensity treatment provided a sense of autonomy. This possibility embodies the essence of PCC as envisioned by Ekman and colleagues (2011), where the ethical imperative to respect and promote patient autonomy is intricately balanced with the commitment to engage the patient’s capacities and personal context [19]. Here, the goal is not merely to empower the patient to manage their condition or pain but to do so within a framework of care that honors their individuality, preferences, and values, ensuring that the care provided is both responsive and responsible.

The second theme uncovered was the reluctance of participants to use pharmacological therapies for pain relief. Patients described their hesitation to use drugs, giving reasons such as nausea, negative cognitive effects, and concern over the risk of addiction. This aligns with findings from other studies, which noted that patients avoided opioid use primarily due to experiencing adverse effects or concerns about the potential for addiction [26, 27]. Around 6% of all patients are at risk of using both NSAIDs and opioids in a persistent and problematic manner after surgery. Hence, TENS is a valuable alternative for patients at an increased risk of short- and long-term side effects of opioid treatment [8, 9].

TENS provides quick pain relief with both rapid onset and offset [15, 17]. This was seen as an advantage compared with oral analgesics that have a slower onset and an unpredictable duration of action, not in the least regarding bothersome adverse effects. Another issue patients raised was that to receive a drug, one must call for help, wait, and rely on healthcare personnel for its administration. This is in contrast to pain management with TENS, wherein a patient has prompt access to the TENS device and can adjust the stimulation intensity according to the severity of their pain, thus, addressing the needs of patients for greater autonomy and rapid pain relief. Olausson et al. (2024) similarly observed that TENS improved patients’ autonomy by facilitating self-management of pain and aiding in pain treatment [28]. In the current study, participants expressed that they would have liked to be able to use TENS at home for ongoing postoperative pain treatment, this likewise underscores patient autonomy that can be fostered using TENS.

Nevertheless, some patients do not get sufficient alleviation of their pain solely with TENS, especially those reporting severe pain intensity when the TENS is initiated, which is a distressing experience for the patients. This group of patients thus needs their pain treatment to be supplemented with conventional analgesics. It is important to recognize that TENS is not appropriate for all patients; therefore, PCC is necessary to identify and respond to the unique needs [19] of patients for pain relief using TENS and/or conventional treatment.

When pain is severe, the stress system in the body is alerted. Since anxiety and pain compound each other, the anxiety relief that opioids provide may also be beneficial for effective pain relief [29].

### Clinical implications

Healthcare providers have a responsibility to listen to patients’ requests and enable their participation in managing their pain after surgery. In the current study, the patients wanted alternative treatments to drugs with fewer side effects. TENS could be implemented in a multimodal pain treatment approach after surgery as a way of promoting control and autonomy for the individual. Not all patients respond to TENS; therefore, a multimodal person-centred approach is appropriate in clinical practice. Further studies are needed on the use of TENS at home as an alternative to conventional treatment with analgesics after surgery.

### Strengths and limitationss

Patients from two different county hospitals were consecutively asked to participate in this interview study, lowering the risk of selection bias. A potential limitation of this study is that participants who are more receptive to complementary therapies may have been more inclined to volunteer, which could introduce a positivity bias in the results. However, all patients who were asked volunteered to participate in the study. Interviews were conducted 7–23 days post-surgery. As the timing of interviews influences participants’ recall of their experiences, recall bias is a potential risk in interview studies, including ours.

Patients reporting both adequate and insufficient pain relief from TENS were included in the interviews. The study included both male and female patients of varying ages. The higher ratio of females to males could be due to cases of cholecystitis.

Addressing transferability, it is essential to contextualize our study’s findings within existing meta-analyses on TENS for postoperative pain management. We acknowledge the limitation that our data may not apply to all surgical scenarios, nevertheless, the use of TENS could still be relevant across various healthcare settings. Our study provides detailed findings as recommended by Houghton et al. [30], and aligns with evidence from meta-analyses on the efficacy of TENS in pain management [17]. To further support the use of TENS in the postoperative care setting, there is a need to evaluate its suitability based on patient factors and surgical procedure characteristics. However, patients should be advised that TENS may not completely alleviate pain without the use of analgesics, and these options should remain accessible. Finally, an ethical limitation of this study is that, although many participants expressed a desire to take the TENS device home, this was not feasible within the current study framework; future studies should consider providing this option to better meet participants’ needs for pain relief.

## Conclusion

This study indicates that patients desire alternatives to drugs for pain control in the postoperative setting. TENS has advantages, such as a rapid onset and offset and supporting patient autonomy, as well as drawbacks, such as not being effective when pain is too severe. TENS could be included within the routine multimodal analgesia framework for person-centred postoperative pain management.

## Electronic supplementary material

Below is the link to the electronic supplementary material.

Supplementary Material 1

Supplementary Material 2

## Data Availability

The data that support the findings of this study are available from the corresponding author upon reasonable request.

## Abbreviations

PACU: Post-anesthesia care unit
PCC: Person-centred care
RCT: Randomized controlled trial
TENS: Transcutaneous electrical nerve stimulation
HFHI TENS: High-frequency, high-intensity transcutaneous electrical nerve stimulation
