# Supplementary material for: Patients´ experiences of TENS as a postoperative pain relief method in the post-anesthesia care unit after laparoscopic cholecystectomy: a qualitative study
Source: BMC Anesthesiol. 2025 Jan 9;25:18. doi: 10.1186/s12871-024-02872-4 (PMC11715503; doi:10.1186/s12871-024-02872-4)
Supplement: Supplementary file 2 — Supplementary Material 2 [file 12871_2024_2872_MOESM2_ESM.docx]

**Supplement 2: Interview guide**

Main question: How have you experienced the treatment with TENS?

• Do you feel that you were involved in your pain management?

• Do you think that the pain relief you received had an effect quickly enough?

• Did you experience any side effects from your pain relief?

• Did you feel that you could gain control over your pain with the help of TENS?

• Do you think that the information provided when the pain relief treatment with TENS was initiated was sufficient?

• What advantages and disadvantages do you see with TENS as postoperative pain relief?

• How have you experienced participating in the study?

Follow-up questions: can you explain what you mean, can you elaborate…
